# Decoding Applications of Artificial Intelligence in Rheumatology

**DOI:** 10.7759/cureus.46164

**Published:** 2023-09-28

**Authors:** Saranya Chinnadurai, Sabarinath Mahadevan, Balakrishnan Navaneethakrishnan, Mahabaleshwar Mamadapur

**Affiliations:** 1 Rheumatology, Sri Ramachandra Institute of Higher Education and Research, Chennai, IND; 2 Rheumatology, O.M. Health Care Clinic, Chennai, IND; 3 Rheumatology, Madras Institute of Orthopaedics and Traumatology (MIOT) Hospital, Chennai, IND; 4 Rheumatology, Jagadguru Sri Shivarathreeshwara (JSS) Medical College, Mysore, IND

**Keywords:** musculoskeletal diseases, ai, autoimmune, arthritis, chatgpt

## Abstract

Artificial intelligence (AI) is not a newcomer in medicine. It has been employed for image analysis, disease diagnosis, drug discovery, and improving overall patient care. ChatGPT (Chat Generative Pre-trained Transformer, Inc., Delaware) has renewed interest and enthusiasm in artificial intelligence. Algorithms, machine learning, deep learning, and data analysis are some of the complex terminologies often encountered when health professionals try to learn AI. In this article, we try to review the practical applications of artificial intelligence in vernacular language in the fields of medicine and rheumatology in particular. From the standpoint of the everyday physician, we have endeavored to encapsulate the influence of AI on the cutting edge of medical practice and the potential revolutionary shift in the realm of rheumatology.

## Introduction and background

Artificial intelligence (AI) has set new frontiers in healthcare in this era of personalized and precision medicine. Over the past decade, research and development in AI have revolutionized health care by transforming the way medical professionals approach various diseases. ChatGPT (Chat Generative Pre-trained Transformer, Inc., Delaware) is the new kid on the block, which has attained attention surpassing social media platforms based on the number of users within a short span of time. The ability of ChatGPT to generate human-like responses quickly became an invaluable tool for medical professionals seeking information on the latest advancements in AI and its applications in medicine [1]. Algorithms, machine learning, deep learning, and data analysis are some of the complex terminologies often encountered when health professionals try to learn AI [2]. This article aims to discuss the merits and demerits of AI in healthcare, in a vernacular way for medical professionals. Also, as rheumatologists, we tried to review the applications of AI in the field of rheumatology.

## Review

What does artificial intelligence mean for healthcare personnel?

Most healthcare professionals and patients are aware of the symptom checker on Google. By simply entering a set of symptoms into a search engine, people can receive a range of potential diagnoses and gain a preliminary understanding of their problem. The symptom checker analyzes the combination and permutation of symptoms from available data to arrive at a list of possible differential diagnoses. Much like a compact AI companion, the symptom checker harnesses the power of algorithms and data analysis to process a multitude of medical information. The benefits of such a tool go beyond mere suspicion of disease diagnosis. It offers a sense of empowerment and autonomy over personal health. Using such tools, individuals can proactively engage with their well-being and initiate informed conversations with healthcare professionals based on the generated insights. Additionally, the symptom checker often serves as a valuable educational resource, enhancing health literacy by promoting awareness of various medical conditions. It also reflects how technology has become deeply intertwined with everyday life, offering an accessible bridge between individuals and medical knowledge. On similar lines, as technology continues to advance, AI works by integrating more comprehensive medical data that could pave the way for smarter symptom checkers and many such tools. More than just an information tool, AI can be customized and optimized for various tasks, depending on the needs. Also, the ability of AI to learn, relearn, and modify based on feedback makes the tool adapt and gain an advantage over time. AI is bound to influence medical care and patient outcomes, both directly and indirectly. Rheumatologists deal with a rare but wide range of disorders involving connective tissue, the musculoskeletal system, and immunology. Such illnesses often receive limited attention among the medical community and the public in general. In such rare specialties, AI could make a meaningful impact. In a real-world, population-based user evaluation study, the novel digital symptom checker ‘Rheumatic?’ was found to be feasible and well accepted by people with rheumatic and musculoskeletal diseases (RMDs) [3].

Medical education

AI may change the way medical education is imparted, accessed, and applied. Integrating AI may help analyze the learning patterns of an individual and tailor the content as per their time and needs. AI may assess the strengths and weaknesses of the professional to create learning modules mimicking real-life scenarios. Also, the medical curriculum may require a revamp of AI to facilitate students dealing with this new tool with confidence. In a study by Civaner M et al., more than 90% of students expressed the gap and need for knowledge regarding the optimal use of AI in medical practice [4]. Medical simulators and 3-D models of the human body supported by AI may provide training options like virtual procedures and surgeries in a risk-free environment before proceeding to real-life situations [5]. In the field of rheumatology, virtual models can be employed for training in procedures like intra-articular injections, lip biopsy for minor salivary glands, muscle biopsy, etc. Apart from training, these virtual models also served as an assessment tool, especially in post-pandemic times [6]. AI may help in reading imaging like CT and MRI by pointing out anatomical abnormalities in an easier, pictorial way. AI can simulate classical patterns like the halo sign and the double contour sign, thereby assisting students in the diagnosis of rare diseases. AI may also act as a virtual mentor, engaging students, answering questions, and summarizing medical literature to date.

Patient education

AI and tools like ChatGPT can provide patients with educational information within a few minutes. The ability to customize information depending on the patient's disease, educational background, and geographical background makes AI score better than the traditional Google search engine. It would help clinicians draft answers to patient’s questions, thereby allowing effective management of time with better consistency. In a study by dermatologists, ChatGPT could provide text consisting of 377.43 ± 60.85 words for a patient education guide on various skin diseases [7]. Virtual assistant tools are already finding their place in chronic illnesses like diabetes [8]. Patients with autoimmune conditions often require lifelong follow-up. Versus Arthritis (Chesterfield, United Kingdom), in collaboration with IBM Watson (New York), has launched a cognitive virtual assistant to provide patient assistance in the field of rheumatology (https://www.ibm.com/case-studies/versus-arthritis). AI can serve as a virtual assistant, clearing patient doubts, triaging patients for consultation, and addressing their concerns in real-time. This will help patients have better control and actively involve them in the management of chronic rheumatological illnesses.

Medical research

AI can analyze the chemical structure of antibiotics and predict the chance of sensitivity or resistance. For instance, Google-powered AI makers MIT (Cambridge, Massachusetts) and McMaster (Elmhurst, Illinois) have analyzed a wide range of chemical molecules against bacteria such as Acinetobacter baumannii in a dish. Now, uploading such data into AI may help in picking up the best combination of such molecules, which will be stable, have better sensitivity, and have the least resistance. Deep learning-guided antibiotic discovery will cut down on cost and time in a significant manner [9]. In similar ways, AI-powered tools can accelerate precision medicine and targeted therapy. In rheumatology, AI can pool data from genomics, proteomics, and metabolomics to stratify patients into homogeneous groups. Such data can eventually be used to optimize candidates for biologics or other drug combinations. Moreover, AI can be employed to test the efficacy and safety of such drugs in virtual patient models. This will cut down on time and cost as well as pave the path to personalized medicine. In rheumatology, such technology can be used for monoclonal antibody development [10].

Medical literature and publication

AI-powered search engines can screen vast databases for relevant articles and analyze and extract patterns, providing meaningful insights about the topic. For editors, AI tools may assess for quality, adherence to guidelines, verifying references, and screening articles for plagiarism. Overall, AI can be potentially used to speed up the publication process [11]. AI tools may also help in multilingual publishing, thereby helping the content reach a wider audience. AI has already been listed as an author in a few publications [12-14]. The scientific and moral issues with listing AI as a co-author are already acknowledged by several journals [15]. AI can potentially replicate and magnify human bias and misinterpretations. The American College of Rheumatology has already expressed concern against listing AI as a co-author in their author guidelines.

AI as a physician assistant

AI can handle initial patient interactions and help triage patients. AI can flag off rare drug interactions and allergies by cross-referencing patient profiles and past medical history. AI can automate medical documentation and cut down physician time on clerical work, which could be directed more toward patient examination. AI can monitor patients in real time and raise alarms for healthcare providers. Rheumatic diseases are often chronic and evolve over time. Often, patients have a history spanning several years and have extensive medical records. AI can summarize meaningful and relevant information from patient medical summaries, thereby significantly reducing the time spent by physicians on analyzing patient records. Such assistance would be of immense help to rheumatologists. AI can also improve remote patient monitoring standards and telemedicine [16].

AI plugins

AI plugins incorporate software into existing applications to add the capabilities of artificial intelligence. Many such FDA-approved devices are already available. RBKnee is one such FDA-approved device to assess the radiographic changes in osteoarthritis. External validation of this AI tool showed good to excellent agreement with the musculoskeletal radiology consultant consensus [17]. More than mere diagnosis, plugins can potentially sum up data from multiple sources. For example, the incorporation of patient activity (sleep, step counts, exercise intensity) with blood sugars and other medical data can predict cardiovascular risk. Such devices would help medical professionals accurately assess individual morbidity and mortality, thereby making meaningful, tailored recommendations for improving the same.

Aid in real-time procedures

By integrating imaging with AI, medical professionals may obtain real-time assistance and feedback in procedures like catheter or stent placement, surgical repair, etc. Such assistance would help in decreasing human errors and improving the success of procedures [18]. A deep learning-based AI tool, Vision Transformer (ViT) was found to be useful in assessing the patterns of microangiopathy on nail fold capillaroscopic (NFC) images. Also, such tools will assist rheumatologists in generating consistent and high-quality NFC reports, especially in the diagnosis of systemic sclerosis [19]. In a study by Gracia Tello et al., a precision rate of 83% and a recall rate of 92.44% were achieved in the identification of capillaries in the nail fold video-capillaroscopic images [20]. Integrating AI into such software can help physicians make better decisions.

Aid in radiology

Radiology is one of the fields that could benefit from incorporating AI. AI can effectively identify or match anatomical variants and assist in diagnosis. AI can perform scoring based on imaging, do measurements instantly, and cut down on the time of a radiologist. AI can analyze high-resolution peripheral quantitative CT images of metacarpophalangeal joints and can differentiate arthritis patients from healthy individuals [21]. A deep learning-based approach using neural networks was employed to define joint shape patterns to classify inflammatory arthritis like rheumatoid arthritis (RA) or psoriatic arthritis [22]. In addition to the diagnosis of RA, machine learning (ML)-based models are useful to assess the disease severity by quantifying synovitis through MRI or ultrasound images [23,24].

Prediction models

AI can predict disease outbreaks and may flag off possible pandemics. At the individual level, AI can analyze complex genetic, epigenetic, geographical, and laboratory data and predict the chances of disease development in those at risk. The classical diagnosis of preclinical rheumatoid arthritis would involve risk scoring using rheumatoid factor and anti-cyclic citrullinated peptide (ACPA) positivity. Often, such models may have a poor predictive value [25]. AI could analyze more complex data such as single nucleotide polymorphisms and predict disease diagnosis in the preclinical stage. ML-based approaches using metabolomics, glycomics, and metagenomics were helpful in differentiating early RA from other arthritis [26,27]. Also, ML-based approaches using the gene profiling of synovial samples were helpful in differentiating RA versus osteoarthritis and early RA versus resolving arthritis [28,29]. Autoimmune diseases are often characterized by remissions and exacerbations. AI may be a useful tool to warn physicians and patients in such scenarios. For example, in rheumatoid arthritis, using data from blood investigations and imaging, AI can also predict relapses [30].

Collaborating in healthcare across regions and time

AI can help in summarizing electronic medical record systems and managing patient data across regions and centers of care. AI technologies can facilitate the organization and accessibility of patient information, allowing rheumatologists across various regions to collaborate and make informed clinical judgments. Such collaborative care among healthcare professionals involved in a patient's treatment can ensure continuity and coordinated management of rheumatic diseases, which often evolve over time.

Limitations of AI

AI may lack human emotions like empathy when delivering critical information. It may not be able to deliver the right information at the right time, considering the patient's social, educational, and emotional standards. AI may also leak confidential information to third parties on the web. This may jeopardize the patient’s trust, ethics, and the doctor-patient relationship. In addition, access to such information by the insurance sector may cause disturbances in the healthcare sector. AI may potentially influence the long-term medical thinking and scientific thirst of the human community. Public misuse of too much technology is not always without dangerous literacy and illusionary patient empowerment [31,32]. AI may not only mimic human intelligence but may also replicate our scientific and societal biases.

## Conclusions

It is as difficult as it is tempting to predict the future of AI. Certainly, AI is here to stay. It is bound to bring revolution in many fields, including medicine. AI is unlikely to replace humans in the art of medicine, where human touch and emotions weigh as much as scientific knowledge. A large amount of absolute correct data is required to be fed into the system before we can let artificial intelligence cross the critical decision-making boundary in the field of medicine. And such data has to be obtained from human intelligence, and interpretation brings us back to square one. Certainly, AI could assist, analyze, and summarize the complex interpretation of data and aid in decision-making. The rheumatology research community is known for increasingly adopting novel techniques like monoclonal antibodies, and AI is no exception, as evidently seen by the number of articles published in this field.
